# Association of chronic obstructive pulmonary disease and hemorrhoids: A nationwide cohort study

**DOI:** 10.1097/MD.0000000000006281

**Published:** 2017-03-10

**Authors:** Lih-Hwa Lin, Justin Ji-Yuen Siu, Po-Chi Liao, Jen-Huai Chiang, Pei-Chi Chou, Huey-Yi Chen, Tsung-Jung Ho, Ming-Yen Tsai, Yung-Hsiang Chen, Wen-Chi Chen

**Affiliations:** a Graduate Institute of Chinese Medicine, School of Chinese Medicine, Graduate Institute of Integrated Medicine, College of Chinese Medicine, College of Medicine, Research Center for Chinese Medicine & Acupuncture, China Medical University, Taichung; b Division of Chinese Medicine, An Nan Hospital, China Medical University, Tainan; c Departments of Urology, Chinese Medicine, Medical Research, and Obstetrics and Gynecology, Management Office for Health Data, China Medical University Hospital; d Department of Urology, Taichung Veterans General Hospital; e Department of Psychology, College of Medical and Health Science, Asia University, Taichung; f Division of Chinese Medicine, China Medical University Beigang Hospital, Yunlin; g Department of Chinese Medicine, Kaohsiung Chang Gung Memorial Hospital and Chang Gung University College of Medicine, Kaohsiung, Taiwan.

**Keywords:** chronic obstructive pulmonary disease, hemorrhoids, lungs, Nationwide Cohort Study, traditional Chinese medicine

## Abstract

According to traditional Chinese medicine (TCM) theory, a specific physiological and pathological relationship exists between the lungs and the large intestine. The aim of this study is to delineate the association of chronic obstructive pulmonary disease (COPD) and hemorrhoids in order to verify the “interior–exterior” relationship between the lungs and the large intestine. A retrospective cohort study is conceived from the National Health Insurance Research Database, Taiwan. The 2 samples (COPD cohort and non-COPD cohort) were selected from the 2000 to 2003 beneficiaries of the NHI, representing patients age 20 and older in Taiwan, with the follow-up ending on December 31, 2011. The COPD cohort (n = 51,506) includes every patient newly diagnosed as having Chronic Obstructive Pulmonary Disease (COPD, ICD-9-CM: 490–492, 494, 496), who have made at least 2 confirmed visits to the hospital/clinic. The non-COPD cohort (n = 103,012) includes patients without COPD and is selected via a 1:2 (COPD: non-COPD) matching by age group (per 5 years), gender, and index date (diagnosis date of COPD for the COPD cohort). Compared with non-COPD cohorts, patients with COPD have a higher likelihood of having hemorrhoids and the age-, gender- and comorbidies-adjusted hazard ratio (HR) for hemorrhoids is 1.56 (95% confidence intervals [CI]:1.50–1.62). The adjusted HR of hemorrhoids for females is 0.79 (95% CI: 0.77–0.83), which is significantly less than that for males. The elderly groups, 40 to 59 years and aged 60 or above, have higher adjusted HRs than younger age groups (20–39 years), 1.19 (95% CI: 1.14–1.26), and 1.18 (95% CI: 1.12–1.24), respectively. Patients with COPD may have a higher likelihood to have hemorrhoids in this retrospective cohort study. This study verifies the fundamental theorem of TCM that there is a definite pathogenic association between the lungs and large intestine.

## 1 Introduction

Chronic obstructive pulmonary disease (COPD) is a type of obstructive lung disease and is related to chronic inflammatory responses to noxious particles such as tobacco smoke and exposure histories. COPD is characterized by irreversible progressive limitations in pulmonary airflow, which causes breathlessness, cough, and excessive sputum production. COPD is preventable and treatable.^[1,2]^ Manifestations of COPD with acute exacerbation include cough, dyspnea, or sputum purulence. Rapid decline of lung function often happens. Many patients have suffered from COPD for years and have endured comorbidities and complications that impacted therapy, prognosis, and quality of life, and then died of the disease or its complications prematurely.^[3,4]^

In the Global Burden of Disease 2004 report published by the World Health Organization (WHO), COPD was the fourth leading cause of death (5.1% of total deaths) in the world, and the ranks of mortality (percent of total deaths) in low-income, middle-income, and high-income countries were the sixth (3.6%), the third (7.4%), and the fifth (3.5%), respectively, in 2004.^[3,4]^ The prevalence of COPD is apparently increasing and should be a crucial issue for future health care providers due to continued exposure of the global population to risk factors for COPD, as well as worldwide demographic trends toward aging populations.^[3,4]^ Moreover, the WHO predicts that morbidity and mortality trends in COPD will increase significantly and will grow to become the third leading cause of death by 2030.^[3,4]^

COPD is a condition that is often associated with increased intraabdominal pressure, and the increased pressure can then be transferred to pressure on the hemorrhoidal tissue, which may then aggravate the condition. In addition, in traditional Chinese medicine (TCM) theory, a specific physiological and pathological relationship exists between these 2 internal organs.^[5]^ Thus, a unique physiological and pathological relationship exists between the lungs and large intestine.^[6]^ The aim of this study is to state and verify the association of COPD and hemorrhoids by investigating the pathogenic abnormality of the lungs and large intestine, respectively.

## 2 Materials and methods

### 2.1 Data source

Longitudinal Health Insurance Database 2000 (LHID2000): The National Health Insurance Administration, Ministry of Health and Welfare, Taiwan, launched a compulsory single-payer National Health Insurance (NHI) program in 1995. The coverage rate has approached more than 99% of Taiwan's 22.9 million residents since 1997.^[7,8]^ The National Health Insurance Research Database (NHIRD) is supervised by the National Health Research Institute, Taiwan, and scientists are authorized to use it for research and medical purposes in Taiwan.

### 2.2 Ethics, consent, and permissions

The LHID 2000 was randomly sampled from beneficiaries of the NHID between 1996 and 2000 and contained all the original claim data of 1 million individuals. LHID 2000 contained all medical expenses, diagnosis, claims of patients between 1996 and 2012, and there was no significant difference in the gender distribution between the patients in LHID 2000 and the original NHIRD. Approval from the institutional review board of China Medical University Hospital was obtained with the number of CMUH104-REC2-115. The identification number of each patient had already been encrypted for privacy protection, and therefore, the informed consent was waived.

### 2.3 Study population

This is a retrospective cohort study and the dataset consists of 2 selected samples (COPD cohort and non-COPD cohort) from the 2000 to 2003 beneficiaries of the NHI representing patients ages 20 and older in Taiwan. The follow-up concluded on December 31, 2011, and the follow-up period lasted for 8 to 11 years. The COPD cohort are patients newly diagnosed as having chronic obstructive pulmonary disease (COPD, ICD-9-CM: 490–492, 494, 496) and have visited the hospital/clinic at least twice as confirmed by LHID 2000. The index date is defined as the new diagnosis date for COPD.

The subject for the comparison group (non-COPD cohort) is a randomly selected patient without COPD. We performed a 1:2 (COPD: non-COPD) matching by age group (per 5 years), gender, and index date (diagnosis date of COPD for the COPD cohort). For the non-COPD cohort, the index date is randomly assigned from years 2000 to 2003 corresponding to the index date distribution of the COPD cohort.

Cases with a history of hemorrhoid before the index date, aged less than 20-years-old, and with incomplete information were excluded.

### 2.4 Outcome

The cohort event is the new diagnosis of hemorrhoid (ICD-9-CM: 455) which occurred after the diagnosis date of COPD in the COPD cohort and the index date in the non-COPD cohort.

### 2.5 Comorbidity

The comorbidities recorded in this study included cirrhosis (ICD-9-CM: 571 and A code: A347), alcoholism (ICD-9-CM: 291, 303, 305.00, 305.01, 305.02, 305.03, 790.3 and V11.3), and heart failure (ICD-9-CM: 428).

### 2.6 Statistical analyses

Two-sample *t*-tests for continuous variables and a chi-square test for categorical variables were used to compare the 2 study groups. The Cox regression model was used to compute the hazard ratio and 95% confidence interval of hemorrhoids co-existing in patients with COPD. The adjusted hazard ratios for COPD were adjusted by age, gender, cirrhosis, alcoholism, and heart failure in Cox proportional hazards regression. The Kaplan–Meier method is used to plot the survival probability; the probability of survival difference between COPD and non-COPD cohort is tested with the log-rank test. All analyses were carried out with SAS statistical software (version 9.4 for Windows; SAS Institute, Inc., Cary, NC). Statistical significance was determined as *P* < 0.05.

## 3 Results

### 3.1 Demographic characteristics

The mean age of patients in the COPD cohort and non-COPD cohort is 53.87 and 52.63 years, respectively. The distribution of age groups is the same in the 2 cohorts: 24.29% for aged 20 to 39 years, 35.79% for aged 40 to 59 years, and 39.92% for aged ≥ 60 years. There are 50.61% men and 49.39% women for both cohorts. Due to our matching process, the distributions of age groups and gender are the same in both cohorts (Table 1).

**Table 1:**
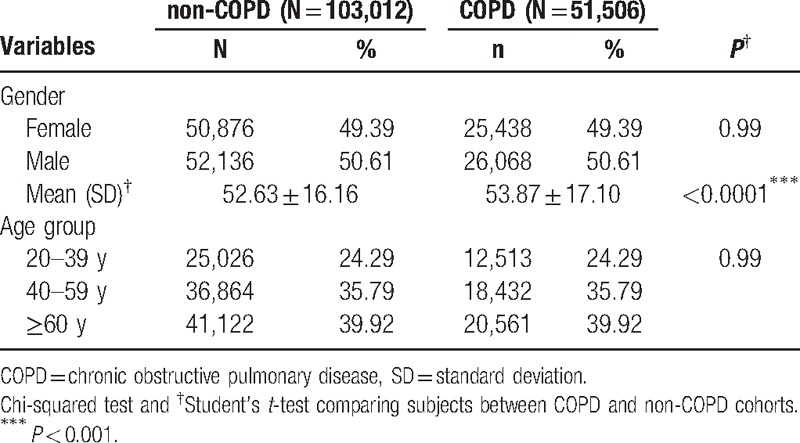
Demographic characteristics of the study population.

### 3.2 The incidence of hemorrhoids and comorbidities

The incidence of hemorrhoid in the COPD cohort (9.38%) is significantly higher than the non-COPD cohort (5.97%) with *P* < 0.001. Moreover, the prevalence of comorbidities in the COPD cohort is significantly higher than the non-COPD cohort: cirrhosis: 26.49% vs 16.48% (*P* < 0.001), alcoholism: 0.44% vs 0.23% (*P* < 0.001), and heart failure: 3.58% vs 1.51% (*P* < 0.001) (Table 2).

**Table 2:**
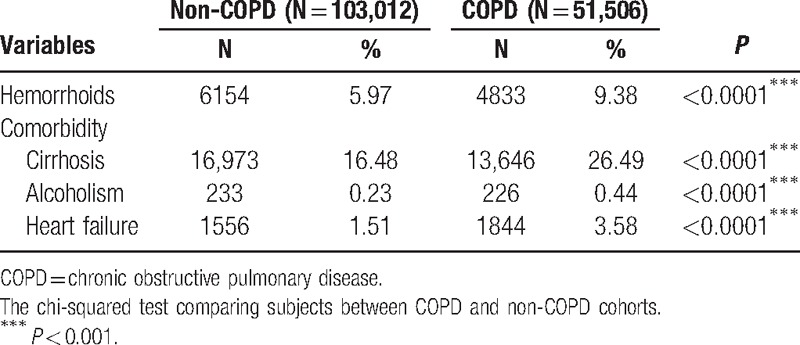
The incidence of hemorrhoids and the prevalence of comorbidities for COPD and non-COPD cohorts.

### 3.3 Hazard ratio (HR)

Table 3 illustrates uni- and multivariate Cox proportional hazard models in the cohort of COPD vs non-COPD. Significant crude hazard ratios of hemorrhoids in the Cox proportional hazard model were COPD (HR: 1.62, 95% CI: 1.56–1.68), female (HR: 0.79, 95% CI: 0.76–0.82), 40 to 59 years (HR: 1.24, 95% CI: 1.18–1.30), more than 60 years (HR: 1.24, 95% CI: 1.18–1.30), cirrhosis (HR: 1.55, 95% CI: 1.49–1.62), alcoholism (HR: 1.66, 95% CI: 1.25–2.22), and heart failure (HR: 1.27, 95% CI: 1.11–1.44). The multivariate Cox model was controlled by 5 co-factors (gender, age, cirrhosis, alcoholism, and heart failure). The model showed that COPD is an independent risk factor for hemorrhoids (HR: 1.56, 95% CI: 1.50–1.62), and cirrhosis exerts a significant independent effect (HR: 1.41, 95% CI: 1.35–1.47).

**Table 3:**
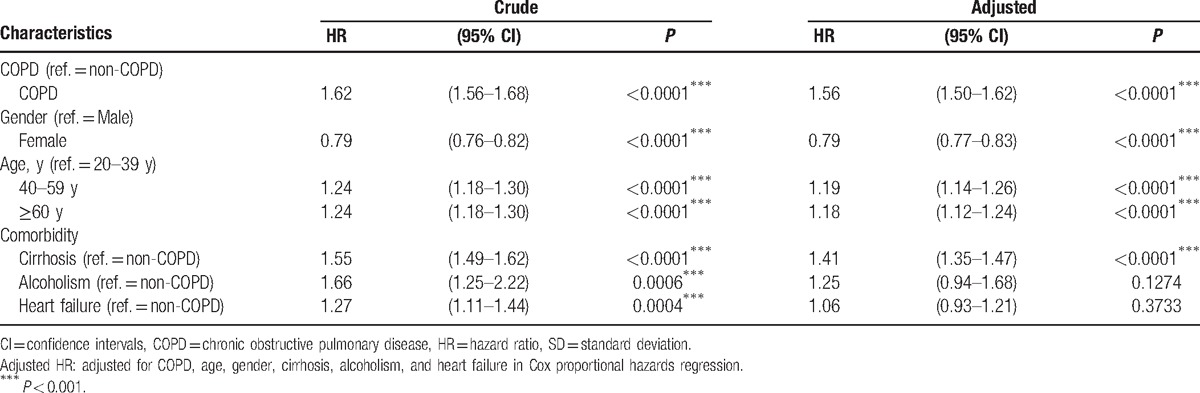
Cox model measured hazard ratio and 95% confidence intervals of hemorrhoids associated with patients in COPD and non-COPD cohorts.

The adjusted HR of hemorrhoids for females (0.79) is significantly less than for males (1.00). Compared with people aged 20 to 39 years, those of aged 40 to 59 years and for aged ≥ 60 years have higher likelihoods of having hemorrhoids, and adjusted HRs were 1.19 and 1.18, respectively (Table 3).

### 3.4 Cohort analysis

The average annual incidence rate of hemorrhoid in the COPD cohort is significantly higher than the non-COPD cohort, 11.45 and 7.08 per 1000 person-years, respectively, and the adjusted HR was 1.56 with *P* < 0.001. Moreover, the average annual incidence rates and HRs of hemorrhoid of both genders and the age groups in the COPD cohort were significantly higher than those in the non-COPD cohort. Females: 9.85 vs 6.31 per 1000 person-years, HR = 1.50 (*P* < 0.001); males: 13.13 vs 7.85 per 1000 person-years, HR = 1.60 (*P* < 0.001); aged 20 to 39 years: 9.65 vs 5.95 per 1000 person-years, HR = 1.58 (*P* < 0.001); aged 40 to 59 years: 12.05 vs 7.36 per 1000 person-years, HR = 1.55 (*P* < 0.001); aged ≥ 60 years: 12.14 vs 7.55 per 1000 person-years, HR = 1.55 (*P* < 0.001). Similarly, the average annual incidence rates and HRs of patients with hemorrhoid and with/without cirrhosis in the COPD cohort were higher than those in the non-COPD cohort: with cirrhosis: 14.37 vs 10.01 per 1000 person-years, HR = 1.45 (*P* < 0.001); without cirrhosis: 10.41 vs 6.52 per 1000 person-years, HR = 1.60 (*P* < 0.001) (Table 4).

**Table 4:**
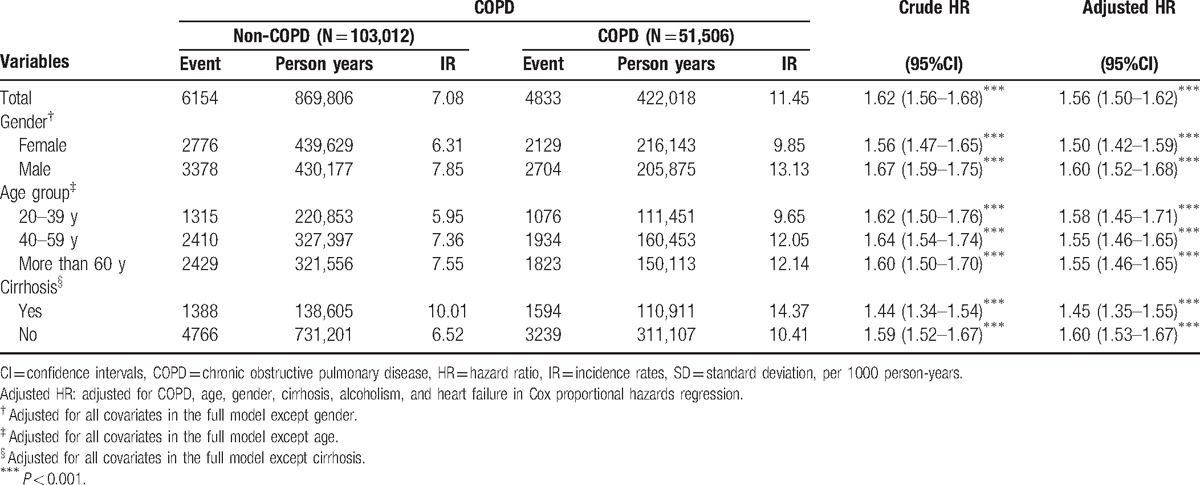
Incidence rates, hazard ratio, and confidence intervals of hemorrhoids for patients with COPD in the stratification of gender and age.

**Figure 1:**
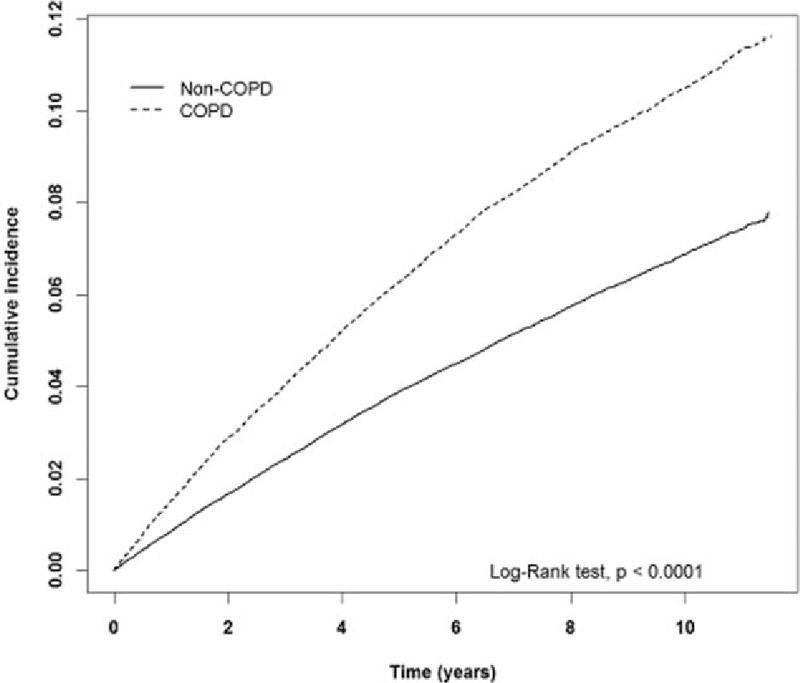
The estimated cumulative incidence of hemorrhoids between the COPD cohort and the non-COPD cohort by Kaplan–Meier analysis. COPD = chronic obstructive pulmonary disease.

**Figure 2:**
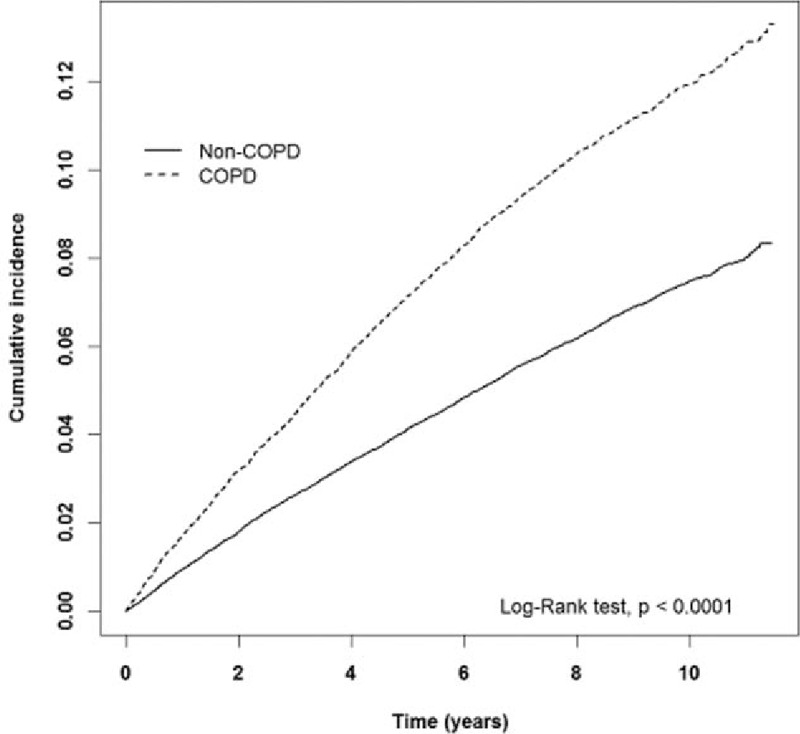
The estimated cumulative incidence of hemorrhoids between the COPD cohort and the non-COPD cohort for males by Kaplan–Meier analysis. COPD = chronic obstructive pulmonary disease.

**Figure 3:**
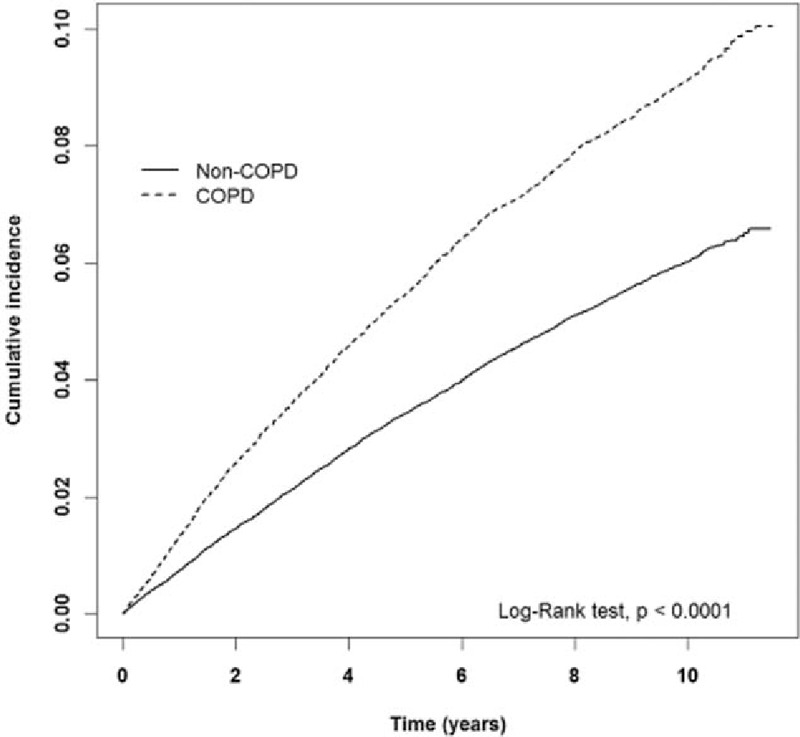
The estimated cumulative incidence of hemorrhoids between the COPD cohort and the non-COPD cohort for females by Kaplan–Meier analysis. COPD = chronic obstructive pulmonary disease.

The estimated cumulative incidence of hemorrhoids in the COPD cohort is obviously higher than that in the non-COPD cohort (*P* < 0.001, log-rank test) (Fig. 1). It is the same as for males (*P* < 0.001, log-rang test) (Fig. 2) and females (*P* < 0.001, log-rank test) (Fig. 3).

## 4 Discussion

In this study, the incidence rates of hemorrhoids are higher in patients with COPD than without COPD. The results provide important information when clinical treatment for hemorrhoid is complicated by its the relationship to pulmonary conditions. The results corresponded to the “lungs and colon have exterior and interior relationships” theory of TCM.^[9]^ According to TCM theory, the lungs represent the respiration system including the nose, pharynx, larynx, trachea, bronchi, bronchioles, and lung parenchyma. According to Huang Di Nei Jing, the lungs open at the nose, which is the outer opening of the respiratory tract, and large particles will be excluded from this first entrance in order to keep the airway clean for ventilation during inhalation. Air will be refined as natural *qi* and impure *qi* by the lungs, and then natural *qi* will reach the viscera and bowels, whereas impure *qi* will be expelled by the lungs.^[10]^

In Western medicine, the primary functions of the large intestine are to store digestive residues and to absorb water. Similarly, in TCM theory, the large intestine conveys digestive residues downward and transforms it into feces. In addition, the large intestine absorbs water from food waste in order to solidify feces. The lungs stand in an interior–exterior relationship with the large intestine.^[11]^ The *lung channel* connects with the large intestine, and vice versa.

COPD is a respiratory disorder and is believed to be largely caused by smoking.^[12]^ Manifestations of COPD ranges from dyspnea, poor exercise tolerance, chronic cough with or without sputum production, and sometimes wheezing to respiratory failure.^[13]^ Patients with COPD with acute exacerbations experiences increased cough, dyspnea, or purulent sputum.^[14]^ There are many comorbidities associated with this disease such as hypercholesterolemia, diabetes mellitus, heart failure, severe obesity, chronic renal failure, and atrial fibrillation.^[15,16]^ Most of these comorbidities can be evaluated by the Global Initiative for Chronic Obstructive Lungs Disease (GOLD) combined risk assessment score (GOLD score).^[17]^ GOLD is a nongovernmental and not-for-profit organization registered with the US tax office.^[13]^ Over the last decade, there were many studies suggesting that COPD is not only a pulmonary problem, but a complex and generalized disorder in the aging population. Increasing evidence points toward chronic inflammation as a key factor in COPD pathogenesis, which can clarify the common pathway linking comorbidities to the disease, such as cardiovascular, skeletal, and nutritional disorders.^[18,19]^

The main hypotheses of hemorrhoid pathophysiology are degenerative change of supportive tissue within the anal cushion, hyperperfusion of hemorrhoidal plexus, and vascular hyperplasia.^[20]^ Elevated anal sphincter pressure is presumed as one of the etiologies, too.^[21]^ Previous studies have suggested that there is significant association between hemorrhoids and obesity.^[22]^ There are many conditions which will contribute to the development of hemorrhoids, such as abdominal straining, lack of fiber intake, prolonged lavatory sitting, constipation, diarrhea, and conditions such as pregnancy, ascites, and pelvic space-occupying lesions that are associated with elevated intra-abdominal pressure.^[23]^

As hemorrhoids are considered to be an aging disorder (regarding COPD), we divided the cohort group and the control group into different age groups and the result of each group showed significant differences in incidence in patients with and without COPD. Regardless of age, patients with COPD have the higher incidence rates of hemorrhoids. We excluded patients aged under 20 years because hemorrhoids seldom develop in patients before age 20.^[24]^

A trend for hemorrhoid development in COPD patients was observed in our study. We assumed that chronic cough and dyspnea which often occurred in COPD patients were related to increased intra-abdominal pressure which caused hemorrhoids. In addition, obesity is related to both hemorrhoids and COPD. Therefore, we recommend regular anorectal examination, self-care for prevention of hemorrhoids, and looking out for anal hemorrhage in COPD patients.

According to TCM theory, *qi*, pertaining to energy fields generated in the body, maintains physiological activities, keeps the body warm, defends the body against pathogens, retains blood flow in the vessels, and helps to nourish the entire body. Each organ has its own *qi* which maintains its physiological activities and disease manifestations. For example, respiration is 1 activity of the *qi* of the lungs, and bowel movement is an activity of the large intestines. The lungs govern *qi* of the whole body, implying that *qi* of the bowel and visceral is derived from the lungs. If lungs cannot supply enough *qi* for the viscera and bowels, it will directly impact the large intestines because of the interior–exterior relationship. According to TCM theory, the lungs not only govern *qi* but also regulate the waterways and water metabolism. Natural *qi* from the lungs goes downward through meridians to provide energy for waterways and to regulate water flow in proper water channels in the body.^[9]^ The lungs provide purified *qi* to the bowels to assist the digestive system to work well, and deploy *qi* downwards in order to make great efforts to convey and transform food wastes through the large intestine, and finally out of the body. Based on this TCM concept, if the lungs become compromised, the large intestine will be considerably hampered in its ability to convey and transform because the lungs cannot supply adequate natural *qi* to aid the large intestine in forcing digestive residues down on a regular basis. Any disturbance of purification and downward passage of *qi* of the lungs will result in the large intestine failing to convey digestive waste, and the bowels may be blocked by refuse materials. Disorders of the large intestine will then occur sequentially.

COPD causes pulmonary airflow restriction, breathlessness, and excessive sputum production. A patient with COPD fails to inspire fresh air, lacks the ability to purify air to become natural *qi*, and provides insufficient essential *qi* and energy for the viscera, especially the large intestine. The large intestine will not have adequate natural *qi* from the lungs to perform normal physiological activities, and consequentially motility disorders of the large intestine, constipations and hemorrhoids will occur.

Hemorrhoids are vascular cushions of mucosa filled with veins, muscle fibers and connective tissue. Hemorrhoids may cause pain, itching, rectal bleeding, or palpable mass in the perianal region.^[12]^ Hemorrhoid is a benign anorectal disorder and is fairly common not only in the elderly, but also in younger people, and may negatively impact a person's quality of life.^[13]^ The exact prevalence of symptomatic hemorrhoid is hard to estimate, because many patients do not visit clinics or hospitals to treat this problem. The prevalence of symptomatic hemorrhoid disease in the United States is estimated 4.4% to 40.0%.^[19]^ The association of COPD and hemorrhoids represent abnormalities of the lungs and large intestine in this study, and there is a pathological relationship between these 2 internal organs.

We used a nation-wide database that contained data from all hospitals, medical centers, and clinics in the country, so it is a heterogeneous database. We only included a patient if the same ICD code of diagnosis was used in at least 2 billing occasions to avoid the problem of erroneous coding. The follow-up period is more than 10 years, which increases the validity of our results. We separated the cohort group and the control group according to age to emphasize that the relationship of COPD and hemorrhoid exists in different age groups.

We could not provide certain details, such as the type, symptoms, severity, and family history of hemorrhoid of individual patients, because of the nature of the NHI database. In addition, hemorrhoids are common during pregnancy and the puerperium. Acute hemorrhoid discomfort was aggravated in pregnant women with pre-existing hemorrhoids,^[25]^ but we did not collect this information. No doubt, smoking is a strong risk factor of COPD, but the relevant information could not be provided due to the nature of the NHI database.

In conclusion, patients with COPD may be at a higher risk of developing hemorrhoids in this retrospective cohort study. This study verifies the fundamental theorem of TCM that the lungs and large intestine have an interior–exterior relationship. To the best of our knowledge, this is the first study and survey about the “lungs and colon have exterior and interior relationships” theory of TCM.
